# Suboptimal Choice in Pigeons: Stimulus Value Predicts Choice over Frequencies

**DOI:** 10.1371/journal.pone.0159336

**Published:** 2016-07-21

**Authors:** Aaron P. Smith, Alexandria R. Bailey, Jonathan J. Chow, Joshua S. Beckmann, Thomas R. Zentall

**Affiliations:** Department of Psychology, University of Kentucky, Lexington, Kentucky, United States of America; Universidad de Chile, CHILE

## Abstract

Pigeons have shown suboptimal gambling-like behavior when preferring a stimulus that infrequently signals reliable reinforcement over alternatives that provide greater reinforcement overall. As a mechanism for this behavior, recent research proposed that the stimulus value of alternatives with more reliable signals for reinforcement will be preferred relatively independently of their frequencies. The present study tested this hypothesis using a simplified design of a Discriminative alternative that, 50% of the time, led to either a signal for 100% reinforcement or a blackout period indicative of 0% reinforcement against a Nondiscriminative alternative that always led to a signal that predicted 50% reinforcement. Pigeons showed a strong preference for the Discriminative alternative that remained despite reducing the frequency of the signal for reinforcement in subsequent phases to 25% and then 12.5%. In Experiment 2, using the original design of Experiment 1, the stimulus following choice of the Nondiscriminative alternative was increased to 75% and then to 100%. Results showed that preference for the Discriminative alternative decreased only when the signals for reinforcement for the two alternatives predicted the same probability of reinforcement. The ability of several models to predict this behavior are discussed, but the terminal link stimulus value offers the most parsimonious account of this suboptimal behavior.

## Introduction

Suboptimal choice, or when animals choose an alternative that leads to less reinforcement than another, has gained attention in part because such counterintuitive results are inconsistent with optimal foraging theory [1]. This theory, emphasizing an evolutionary basis of behavior, suggests that animals will prefer alternatives that maximize energy intake, or reinforcement, while minimizing effort. However, various choice procedures have demonstrated conditions in which animals prefer stimulus alternatives that lead to as little as 10% of the reinforcement as the optimal alternative [2–7]. Further, this suboptimal behavior may resemble and serve as a model for human gambling [8–10], in which humans regularly engage in behavior that normally results in a net loss of resources [11].

**Fig 1:**
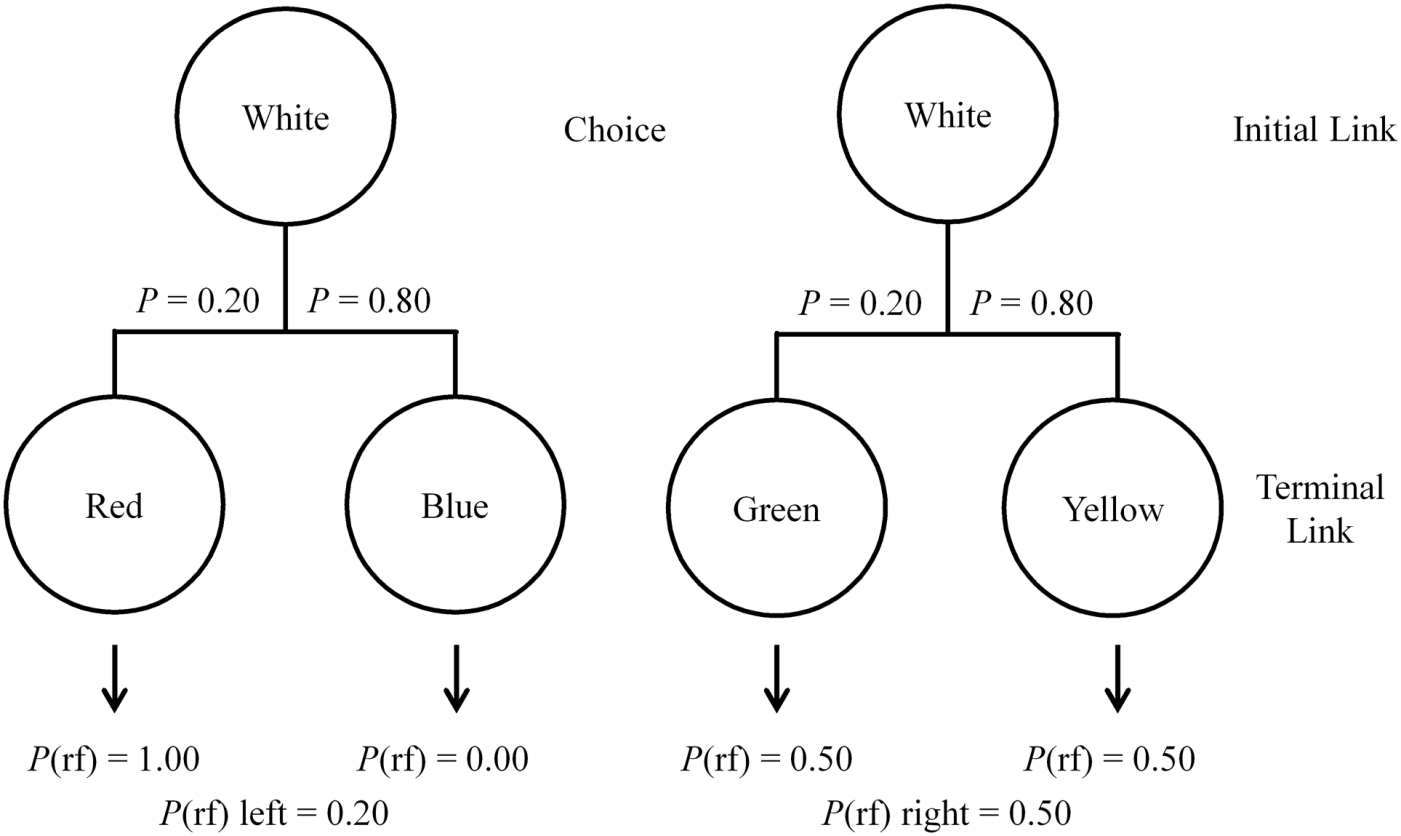
Design of the 4-stimulus suboptimal choice experiment using a spatial discrimination between two white keys. The left alternative shows possible outcomes for choosing the Discriminative alternative while the right alternative shows the Nondiscriminative outcome.

The design of such an experiment is illustrated in Fig 1. With this procedure, pigeons choose between two initial link stimuli. Choice of the left initial link stimulus leads, 20% of the time, to a red stimulus for 10 s that always signals reinforcement but leads to a blue stimulus 80% of the time that signals the absence of reinforcement. Thus, the left initial link predicts 20% reinforcement overall. Alternatively, choice of the right initial link stimulus always leads to a stimulus (20% of the time to a green stimulus and 80% of the time to a yellow stimulus) that predicts reinforcement 50% of the time. Thus, even though the right choice alternative predicts 2.5 times as much reinforcement, Stagner and Zentall [6] found that pigeons show a very strong preference for the left, suboptimal alternative. We refer to this procedure as the 4-stimulus design because of the number of terminal link stimuli. Using this and other similar 4-stimulus procedures [2, 12, 13], large and consistent preferences for the suboptimal alternative have been found.

It appears from this research that pigeons prefer the terminal link stimulus with the greatest ability to predict reinforcement (100%) despite the fact that it occurs only 20% of the time. This finding suggests that the signal for reward omission (the S-) that occurs 80% of the time has little conditioned inhibitory effect. Indeed, Laude, Stagner, and Zentall [14], using a compound cue test that combined the signal for reinforcement and the signal for the absence of reinforcement, found early in training the S- showed some conditioned inhibitory strength that weakened with added training. Additionally, using an unavoidable diffuse houselight as the S- signal [15] and varying the amount of time spent in the presence of the S- signal [16] showed little effect on suboptimal preference. Thus, it appears that the signal for nonreinforcement has little effect on choice and strengthens the conclusion that the primary determinant of initial link choice is the predictive value of the conditioned reinforcers [12].

**Fig 2:**
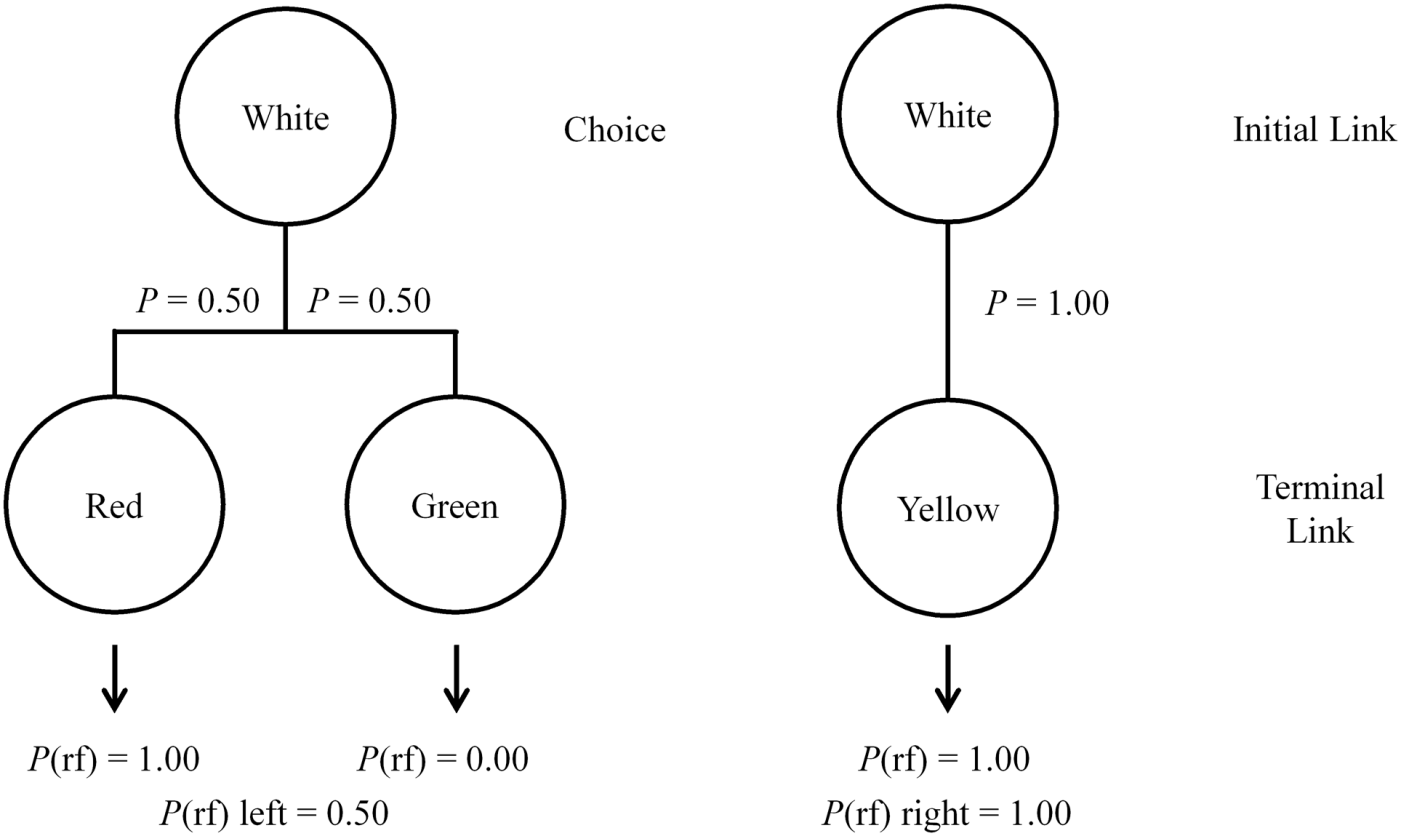
Design of the 3-stimulus suboptimal choice experiment using a spatial discrimination between two white keys. The left alternative shows possible outcomes for choosing the Discriminative alternative while the right alternative shows the Nondiscriminative outcome.

Early research that first described suboptimal choice [3, 5, 17–19] could be interpreted as consistent with this conclusion. In those experiments, a design similar to that shown in Fig 2 (denoted the 3-stimulus design) was used in which the optimal (right) alternative led to a stimulus that always predicted reinforcement, whereas the suboptimal (left) alternative led, 50% of the time, either to a stimulus that was always followed by reinforcement or a different stimulus that was never followed by reinforcement. With this procedure, however, inconsistent suboptimal preferences were typically found with what often appeared to be large individual differences. If the value of the terminal link conditioned reinforcer determines the initial link preference, the pigeons in this earlier research should have been indifferent between the two alternatives because both had a conditioned reinforcer that perfectly predicted reinforcement.

Smith and Zentall [20] concluded that these inconsistent results may have resulted from the fact that the optimal and suboptimal alternatives were signaled by their spatial location alone. The problem with this use of a spatial initial link discrimination is that when pigeons are indifferent between two alternatives they often revert to spatial biases that may have given the impression of a strong preference for that alternative. To test this hypothesis, Smith and Zentall [20] used the 3-stimulus design illustrated in Fig 2 but employed a visual discrimination in the initial link stimuli signaling optimal and suboptimal alternatives that randomly changed locations between trials. Under these conditions, if the pigeons were indifferent between the two alternatives and developed a spatial bias, initial link preference would be at 50% due to their changing locations. Consistent with their hypothesis, the pigeons showed indifference between the initial link alternatives. This indifference was also not a result of a failure to discriminate between the initial link cues as further manipulation of the values of the conditioned reinforcers continued to accurately predict initial link preferences.

Although other factors may contribute to suboptimal choice (e.g., [3, 17, 18]), the results reported by Smith and Zentall [20] indicate that the conditioned reinforcement value of the terminal links indeed play a critical role in choice preferences that they termed the stimulus value hypothesis [8, 12, 20]. A similar conclusion was reached by Mazur (e.g. [3, 21]), however interpretation of this research is made difficult because, as described above, initial link spatial discriminations were used. A further extension of the stimulus value prediction is that, paradoxically, the relative frequencies of the predictive stimulus should play little role in its preference. Indeed, this conclusion was supported by recent research with starlings in which decreasing the frequency of the S+ stimulus showed little decline in initial link preference [7], but has yet to be demonstrated in pigeons.

**Fig 3:**
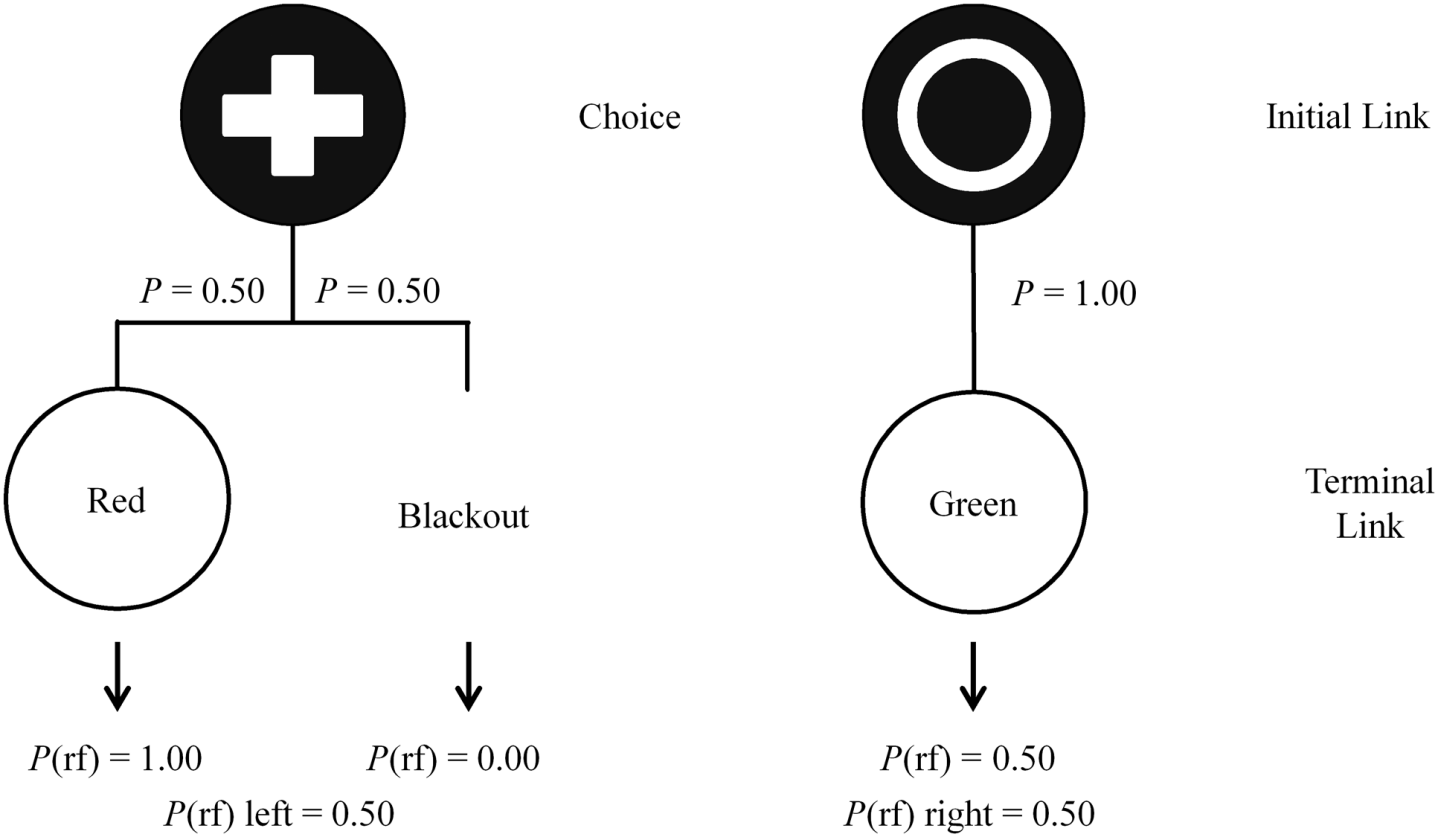
Design of the 2-stimulus suboptimal choice experiment using a visual discrimination. The left alternative shows possible outcomes for choosing the Discriminative alternative while the right alternative shows the Nondiscriminative outcome.

Thus, the purpose of the present experiments was to extend the research reported by Smith and Zentall [20] by systematically varying the frequency of the conditioned reinforcers. Additionally, because of the evidence that the S- signal has been found to have little effect on initial link choice [14–16], the present study used a simplified 2-stimulus design involving only two terminal link stimuli with one following each initial link alternative (see Fig 3; see also [3]). In Experiment 1, the reinforcement rates associated with the two alternatives were initially equal with choice of the Discriminative option leading, 50% of the time, to a signal for either reinforcement or blackout for 10 s. Conversely, choice of the Nondiscriminative alternative always led to a terminal link stimulus that signaled reinforcement 50% of the time. If the stimulus value hypothesis is correct, these conditions should result in preference for the Discriminative alternative. Then, in subsequent phases, reducing the frequency of the Discriminative alternative’s predictive stimulus (and its reinforcement rate) should not reduce this preference even when it becomes suboptimal.

## Experiment 1

### Method

#### Subjects

Ten pigeons (five Homing pigeons and five White Carneau) approximately 8–12 years old originally purchased from the Palmetto Pigeon Plant (Sumter, SC) with previous experience in probabilistic choice tasks were used in the experiment. Subjects were housed in individual cages measuring 28 × 38 × 30.5 cm and maintained at 80%-85% their free feeding weight with free access to grit and water on a 12:12 light-dark cycle (lights off at 7 pm).

#### Ethics statement

All research was approved by the University of Kentucky Institutional Animal Care and Use Committee (Protocol 01029L2006).

#### Apparatus

The experiment was conducted in a standard LVE/BRS (Laurel, MD) chamber measuring 56 × 42 × 37 cm. The pigeons responded to a panel with three square keys approximately 24 cm above the floor, 2.6 cm across, and 1.5 cm apart. The center key was not used in these experiments. A 12-stimulus inline projector (Industrial Electronics Engineering, Van Nuys, CA) behind each key projected one of four stimuli (red, green, plus on a dark background, or circle on a dark background) onto the response keys. A center mounted feeder was located 9 cm beneath the keys that, when raised, was illuminated by a V 0.04-A lamp and allowed access to mixed grain. White noise was generated from outside the chamber, and a computer in an adjacent room controlled the experiment using Med-PC IV [22] with a 10-ms resolution.

#### Procedure

Subjects first trained on an autoshaping procedure. One of the four stimuli were presented on the left or right response key and, after either 30 s or a response to the key, the stimulus was turned off and the feeder was raised for 2 s. Sessions consisted of 60 trials (15 reinforcements per stimulus, counterbalanced across locations). Training continued until two consecutive sessions in which a peck was made 95% of the time.

The procedure (see Fig 3) consisted of both forced and free choice trials separated by a 10-s intertrial interval. On free choice trials, concurrently presented initial link alternatives of a plus and circle on a dark background appeared randomly on either side key (counterbalanced for spatial location across trials). A response to either initial link stimulus extinguished both stimuli. Choice of the Discriminative alternative resulted in either the illumination of the predictive terminal link stimulus (red or green) for 10 s that was always followed by reinforcement or a 10-s blackout period (during which the chamber was dark) 50% of the time. Choice of the Nondiscriminative alternative was always followed by the nonpredictive terminal link stimulus for 10 s but was followed by reinforcement only 50% of the time. Thus, the two alternatives each predicted reinforcement 50% of the time but the terminal link stimulus signaling that reinforcement differed. We refer to the stimulus following choice of the Discriminative alternative the predictive stimulus whereas the stimulus following choice of the Nondiscriminative alternative as the nonpredictive stimulus. Forced choice trials were identical to free choice except only one initial link alternative appeared randomly on either side key, forcing the subject to experience the contingencies associated with that alternative. Sessions consisted of 72 trials, 24 free and 48 forced, with initial and terminal link stimuli counterbalanced across subjects. A stability criterion of at least 15 sessions in which there was no visual or statistically significant trend as defined by a non-zero slope of a line fit through the last 5 sessions was used resulting in 25 sessions of training.

In Phase 2, the probability of the appearance of the predictive stimulus was reduced from 50% to 25%. Training continued again until stability was reached at 25 sessions. In Phase 3, this probability was further reduced to 12.5% with training to stability at 16 sessions. To ensure proper counterbalancing between stimuli and spatial locations, the number of trials in Phase 3 was reduced to 56, 24 free and 32 forced trials. The probability of reinforcement associated with the Nondiscriminative alternative remained at 50% thus making the choices between 25% or 12.5% against 50% reinforcement in Phases 2 and 3, respectively.

#### Data analysis

Choice data were examined using linear mixed effects models in JMP over all sessions of training with subject, phase, and session as factors. Subject was treated as a nominal random factor, phase as a nominal fixed factor, and session as a nominal continuous factor. Latencies to choose and number of pecks to the terminal link stimuli during forced choice trials were also analyzed using linear mixed effects over the average of the last five sessions of training with subject, phase, and trial type (Discriminative or Nondiscriminative) as factors. Subject was again treated as a random nominal factor, phase as a fixed continuous factor, and trial type as a fixed nominal factor. Reported means and standard errors represent the average from the last five sessions of training in each phase.

### Results

#### Choice data

**Fig 4:**
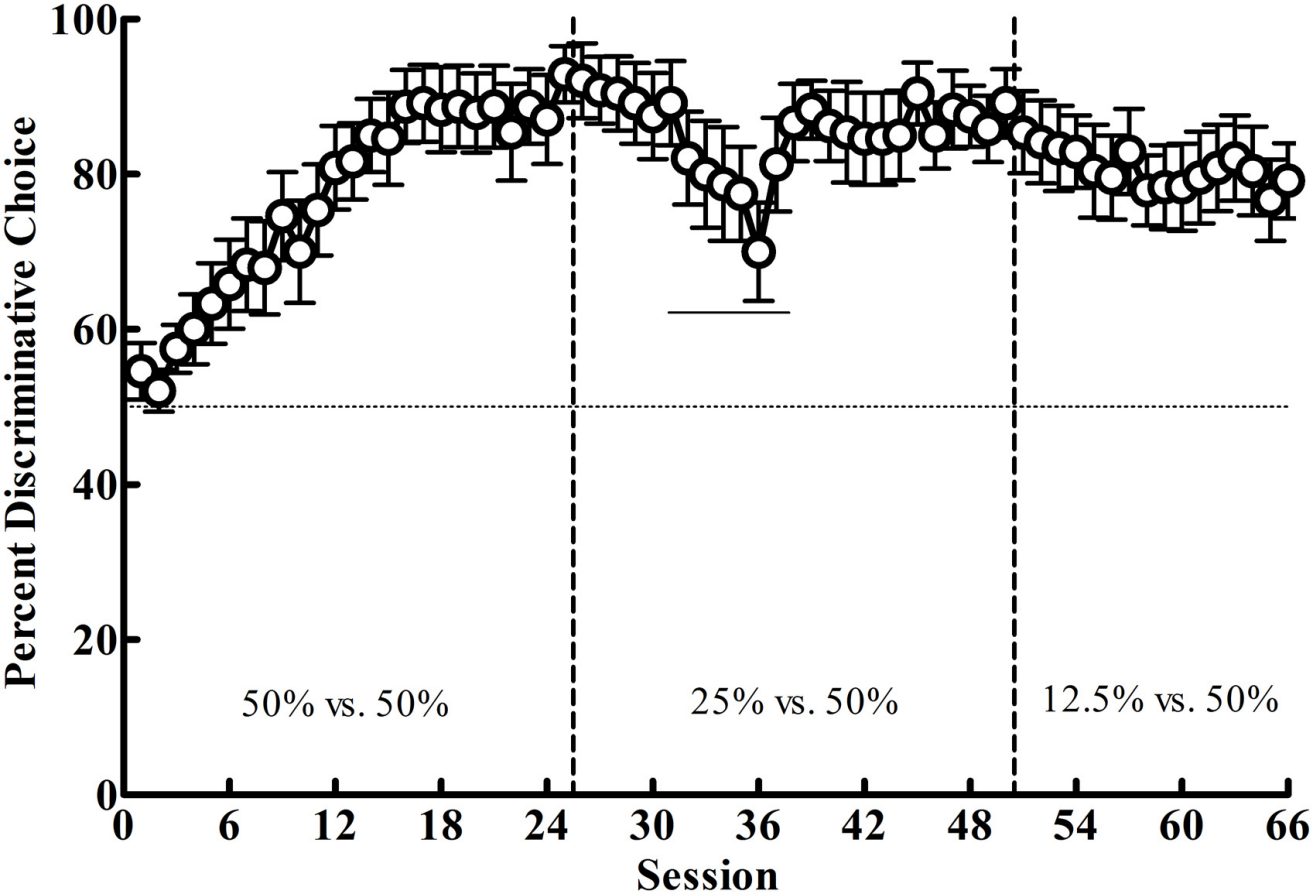
Experiment 1: Mean percentage choice (± SEM) of the Discriminative alternative as a function of session for Phases 1–3. Vertical lines indicate phase changes, the horizontal line indicates indifference between the two altrnatives (50%), and the solid horizontal line indicates sessions where the left response key intermittently malfunctioned.

In Phase 1 (50% vs. 50%), where reinforcement was equated between the alternatives, the pigeons showed a strong preference for the Discriminative alternative (*M* = 88.58; *SEM* = 5.14; see Fig 4). In Phase 2, when the reinforcement associated with the Discriminative alternative was reduced to 25% (25% vs. 50%), a malfunctioning response key for several sessions resulted in a decreased preference for the Discriminative alternative. When the key was repaired, this preference returned to its previous level (*M* = 87.17; *SEM* = 4.37). Thus, there appeared to be little effect of the decrease in reinforcement rate on initial link preference for the majority of pigeons. Finally, in Phase 3, when the reinforcement rate for the Discriminative alternative was again cut in half to 12.5%, there appeared to be a small decrease in preference for the Discriminative alternative by the last five sessions of training (*M* = 79.83; *SEM* = 5.37). To quantify these apparent trends, the linear mixed effects model revealed significant effects of session, *F*(1, 9.58) = 14.07, *p* = .004, and a Phase × Session interaction, *F*(2, 17.7) = 20.06, *p* < .001. Post-hoc analyses indicated a significantly increasing slope in choice of the Discriminative alternative in Phase 1, *p* < .001, but slopes not significantly different from zero in Phases 2 and 3, *p ≥* .209. Thus, the data suggest that although there were individual differences (see *Stimulus Value* section below), the pigeons preferred the Discriminative alternative when the reinforcement rate between the alternatives was equal and they showed no reduction in preference despite reducing its reinforcement rate by 75% in Phase 3. Finally, Table 1 shows the average food earned from each alternative across the last five sessions of each phase.

**Table 1:** Average food reinforcers earned from each alternative across the last five sessions of Phases 1–3 in Experiment 1.

	50% vs. 50%		25% vs. 50%		12.5% vs. 50%	
Subject	Disc. alt.	Nondisc. alt.	Disc. alt.	Nondisc. alt.	Disc. alt.	Nondisc. alt.
20	23.5	12.5	12.0	12.1	4.9	8.5
135	24.0	12.0	11.4	13.3	3.7	13.2
245	20.1	15.9	11.3	13.4	4.4	10.4
254	23.6	12.4	10.2	15.6	4.4	10.6
257	23.7	12.3	11.9	12.2	5.0	8.2
1056	22.6	13.4	11.0	14.1	3.8	13.0
4074	18.5	17.5	9.8	16.4	4.0	12.1
19227	22.5	13.5	11.0	14.1	4.2	11.2
19229	23.9	12.1	12.0	12.0	4.8	8.7
22642	23.9	12.1	11.9	12.2	4.9	8.3
Mean	22.63	13.37	11.23	13.54	4.40	10.42
Proportion	0.629	0.371	0.453	0.547	0.297	0.703

#### Latency & response rate data

**Fig 5:**
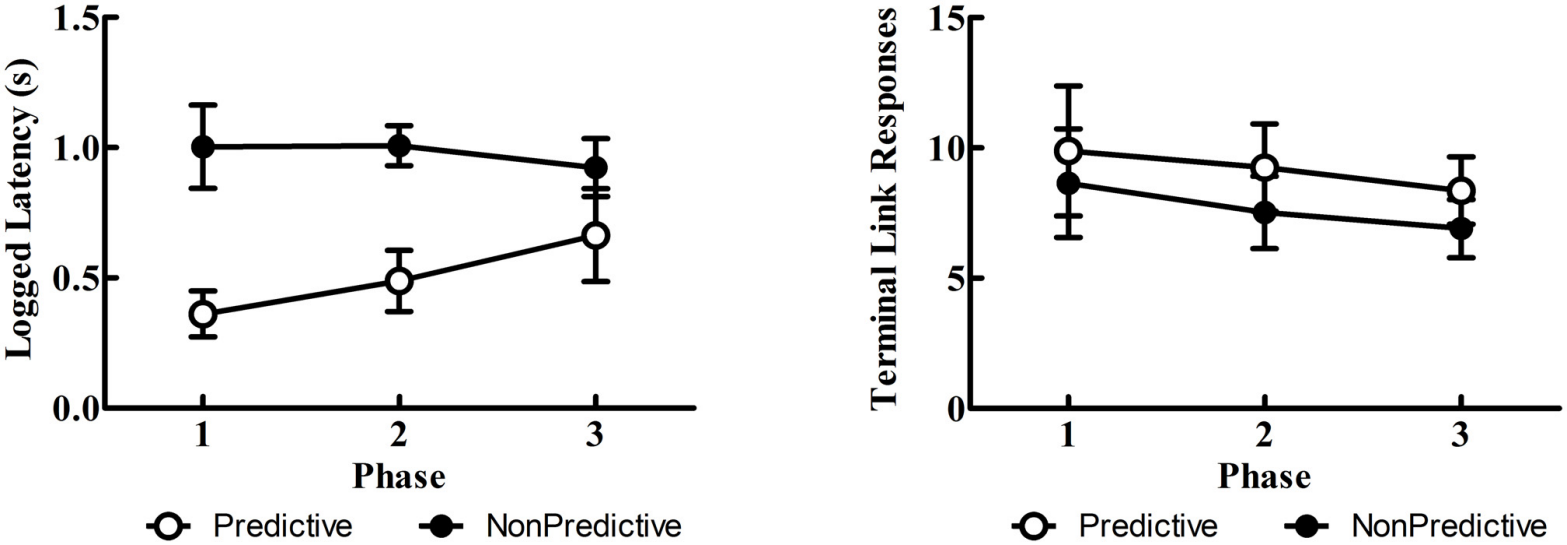
Experiment 1: Mean logged latencies (± SEM) to choose (left panel) and terminal link responses (right panel) for Phases 1–3.

Overall, latencies to choose the Discriminative alternative were generally shorter than the Nondiscriminative alternative (see Fig 5). Additionally, across phases, latencies to choose the Discriminative alternative appeared to increase as their reinforcement rates decreased. The linear mixed effects model, however, revealed only a significant effect of trial type, *F*(1, 9) = 134.61, *p* < 001, indicating that latencies to respond to the Discriminative alternative were significantly shorter; the Trial Type × Phase interaction did not reach statistical significance, *p* = .053. Response rates to the terminal link stimuli during forced choice trials (see Fig 5) were quite similar. There appeared to be a small increase in response rates to the Discriminative alternative’s predictive stimulus, however the linear mixed effects model revealed no significant effect of trial type, *p* = .052.

### Discussion

The purpose of Experiment 1 was to further test the stimulus value hypothesis by systematically reducing the frequency of the Discriminative alternative’s predictive stimulus (and reinforcement rate) compared to an alternative that did not have as good a predictive stimulus but provided more food overall. If the stimulus value hypothesis is correct, the Discriminative alternative should be preferred initially and remain preferred despite a reduction in its reinforcement rate. In support of this prediction, the Discriminative alternative was highly preferred in Phase 1. When that alternative was devalued by reducing the amount of primary reinforcement associated with it, there was no significant change in preference for it in Phases 2 and 3. Further, even though there was a key malfunction in Phase 2 (see Fig 4, 25% vs. 50%), once it was repaired the preference for the discriminative alternative returned to the level it was in Phase 1. These findings support the results found with starlings [7], the general conclusions of Mazur [3, 21], and the predictions made by Smith and Zentall [20]. Additionally, these results indicate that omitting the signal for nonreinforcement following choice of the Discriminative alternative does not appear to change the preference for the suboptimal alternative compared with studies using a signal (S-) for the absence of reinforcement (e.g. [6]). Finally, although one might argue that carryover effects from Phase 1 may have maintained the preferences found in Phases 2 and 3, previous research has shown similar effects when the probability of the predictive stimulus was only 20% from the outset of training (e.g., [6]), suggesting that carryover effects are not responsible for these results.

The shorter latencies to respond to the Discriminative alternative are also consistent with its greater preference. While only qualitative, this finding supports sequential choice theories that have posited differential choice latencies are indicative of choice preferences [7, 23]. Also of interest was the fact that, as the Discriminative alternative was devalued in Phases 2 and 3, latencies to respond to the Discriminative alternative began to rise, while latencies to the Nondiscriminative alternative remained relatively stable. Although the interaction was not statistically significant, the finding that devaluing an alternative selectively affected only latencies to that alternative is consistent with sequential choice theories. Future research using latency of response to the alternatives may be able to reduce the variance of those latencies by requiring an orienting response to the center response key prior to presentation of the initial choice alternatives. Response rates to the terminal link stimuli, however, did not appear to reflect choice preferences despite a tendency for increased pecking to the Discriminative alternative’s terminal link. Indeed, one might posit that, due to its reduced frequency, the appearance of the Discriminative alternative’s clear signal for reinforcement might elicit greater behavioral activation as has been suggested elsewhere [24], yet the present study did not find evidence of this (perhaps due to a ceiling effect). Similar to previous accounts, the present results suggest that response rates are not always indicative of an alternative’s value (see [25] for a discussion).

In Experiment 1 we tested predictions made by the stimulus value hypothesis [8, 12, 20] by decreasing the frequency of the Discriminative alternative’s predictive stimulus. Alternatively, one could also test the stimulus value hypothesis by increasing the predictive value of the Nondiscriminative alternative’s terminal link stimulus. According to the stimulus value hypothesis, if the contingencies start as in Phase 1 with 50% reinforcement for both choices and then the predictive value of the Nondiscriminative alternative’s terminal link is increased to 100%, preference should remain for the Discriminative alternative until the terminal links have equal predictive value (100%). At this point, according to the stimulus value hypothesis, there should be indifference (50% preference) between the two alternatives because conditioned reinforcers following both alternatives should predict reinforcement equally [20].

## Experiment 2

### Method

#### Subjects and apparatus

The subjects and apparatus were the same as in Experiment 1.

#### Procedure

Experiment 2 began immediately after Experiment 1 by returning to the stimulus values of Phase 1 in Experiment 1 (50% vs. 50% reinforcement; see Fig 3) but reversed the associations between the initial and terminal link stimuli. The reversal was trained to the same criterion in Experiment 1, lasting 55 sessions.

In Phase 2 of Experiment 2 the predictive value of the Nondiscriminative alternative’s terminal link stimulus was increased to 75% reinforcement. That is, it appeared 100% of the time but the probability of reinforcement after its appearance increased from 50% to 75%. Training continued until criterion at 21 sessions. In Phase 3, the predictive value of the Nondiscriminative alternative’s stimulus was further increased to 100%. This equated the two terminal link stimuli in terms of their predictive validity (each signaled reinforcement 100% of the time), however the Nondiscriminative alternative provided twice as much reinforcement (100% vs. 50%). Choice of the Discriminative alternative continued to result in presentation of its predictive stimulus 50% of the time. Although both choice alternatives and terminal link stimuli were discriminative and predictive in this phase, the previous nomenclatures were maintained for consistency. Training continued until criterion which occurred at 23 sessions.

### Results

#### Choice data

**Fig 6:**
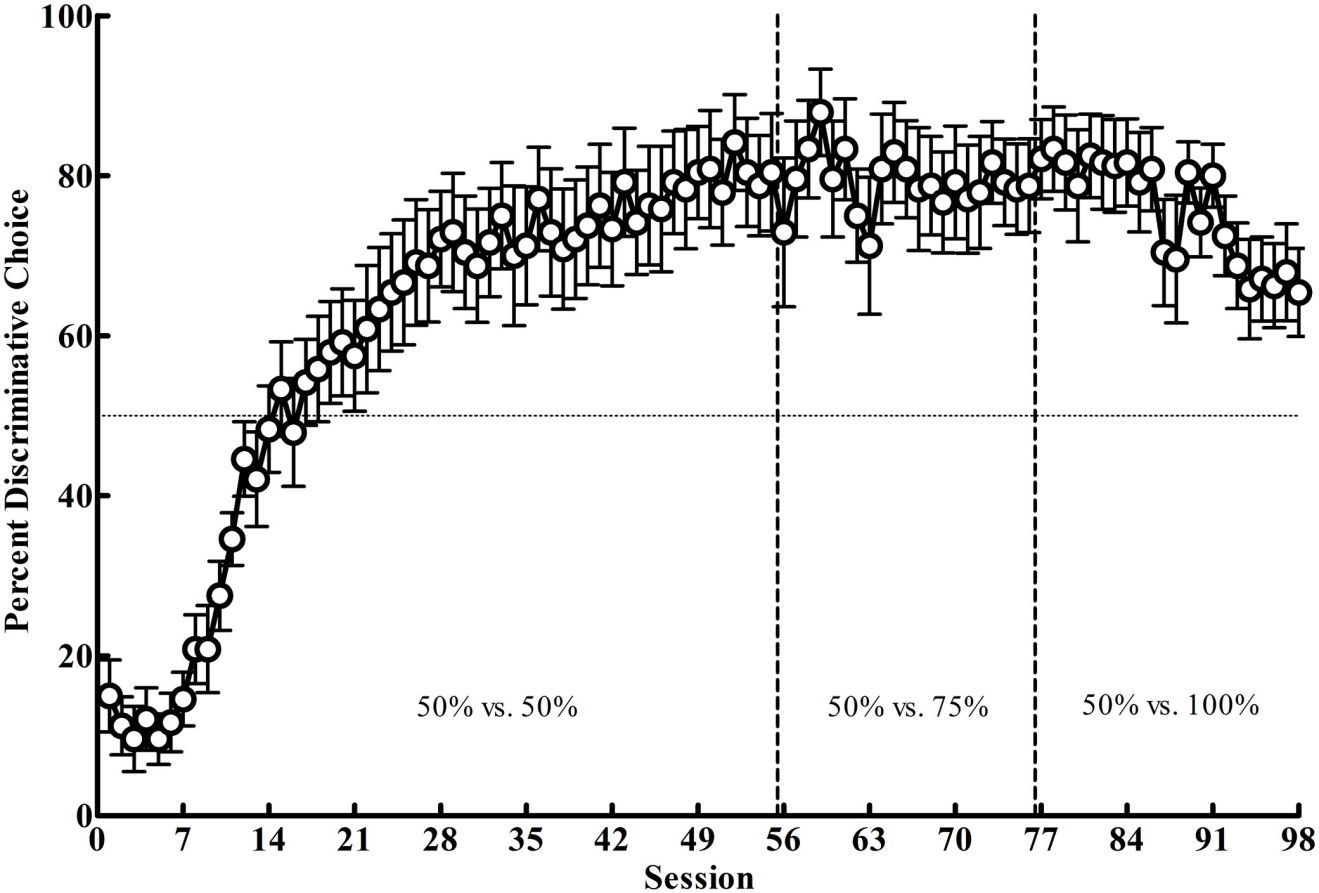
Experiment 2: Mean percentage choice (± SEM) of the Discriminative alternative as a function of session for Phases 1–3. Vertical lines indicate phase changes while the horizontal line indicates chance (50%).

Given that the contingencies of Experiment 2 involved a reversal from the contingencies of Experiment 1, the pigeons initially chose the Nondiscriminative alternative but quickly developed a preference for the Discriminative alternative with added training (*M* = 80.33, *SEM* = 6.59), replicating the preference found in Phase 1 of Experiment 1 (see Fig 6). In Phase 2, increasing the predictive validity of the Nondiscriminative alternative’s terminal link stimulus to 75% produced little change in preference (*M* = 79.17, *SEM* = 5.80). However, in Phase 3, further increasing its predictive value such that both terminal link stimuli were of equal value (100%) did produce a drop in the preference for the Discriminative alternative, at least for some pigeons (*M* = 66.50, *SEM* = 5.65). These trends were confirmed with the linear mixed model which revealed a significant effect of phase, *F*(2, 18.02) = 25.77, *p* < .001, and Phase × Session interaction, *F*(2, 20.62) = 25.88, *p* < .001. The interaction resulted from a significantly increasing slope in Phase 1 reflecting the reacquired preference for the Discriminative alternative, no significant trend in Phase 2 with the increase in the nonpredictive stimulus’ predictive validity to 75%, and a significant decreasing slope in Phase 3 when the reinforcement rate was further increased to 100%. Additionally, post-hoc comparisons indicated that all slopes were significantly different from each other, *ps* < .05. A one-sample *t*-test on the last five sessions from Phase 3 also confirmed that the pigeons’ preference remained significantly above chance, *t*(9) = 3.13, *p* = .012. The pigeons’ average food earned for each alternative in the last five sessions of training in each of the three phases is shown in Table 2.

**Table 2:** Average food reinforcers earned from each altenrative across the last five sessions Phases 1–3 in Experiment 2.

	50% vs. 50%		50% vs. 75%		50% vs. 100%	
Subject	Disc. alt.	Nondisc. alt.	Disc. alt.	Nondisc. alt.	Disc. alt.	Nondisc. alt.
20	18.0	18.0	18.1	26.8	18.0	36.0
135	22.2	13.8	21.1	22.4	20.7	30.6
245	23.6	12.4	23.7	18.4	16.8	45.6
254	21.9	14.1	22.9	19.6	20.2	31.6
257	23.9	12.1	23.7	18.4	24.0	24.0
1056	17.4	18.6	18.2	26.7	19.4	33.2
4074	20.6	15.4	21.4	21.9	20.8	30.4
19227	20.8	15.2	20.6	23.1	20.8	30.4
19229	24.0	12.0	22.1	20.8	20.8	30.4
22642	24.0	12.0	23.2	19.2	18.3	35.4
Mean	21.64	14.36	21.50	21.75	19.98	32.76
Proportion	0.601	0.399	0.497	0.503	0.379	0.621

#### Latency & response rate data

**Fig 7:**
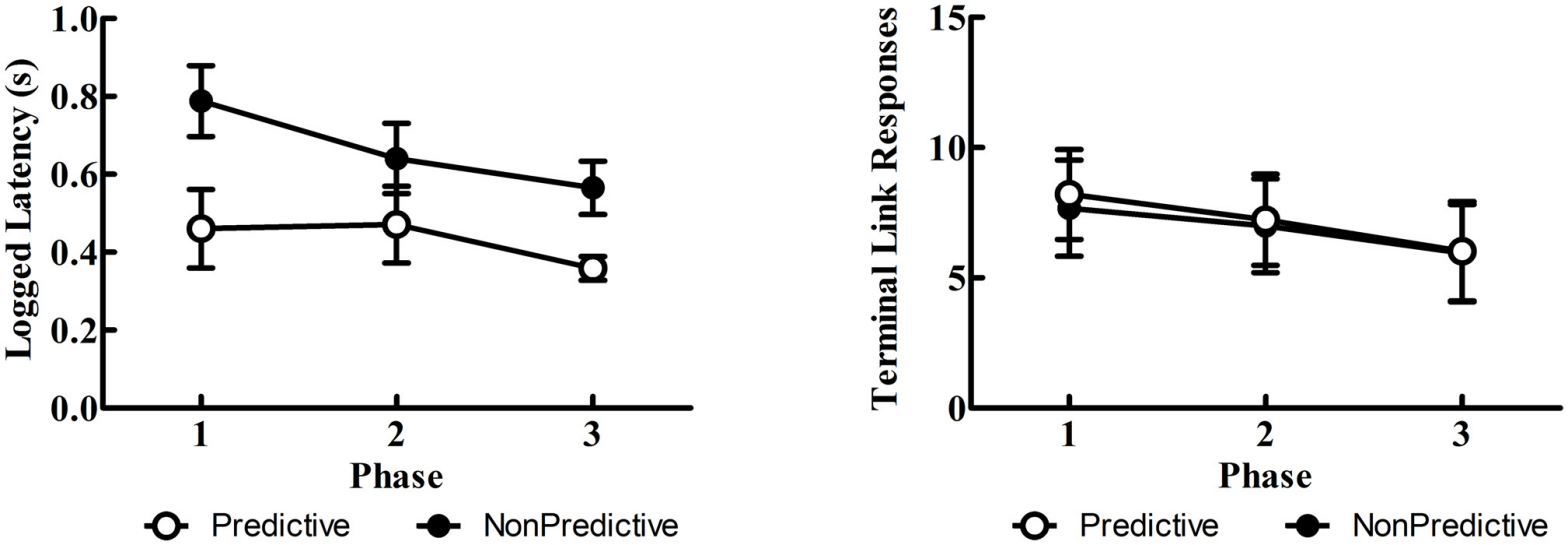
Experiment 2: Mean logged latencies (± SEM) to choose (left panel) and terminal link responses (right panel) to the terminal link stimuli for Phases 1–3.

Latency to respond to the initial link alternatives again showed shorter latencies for the Discriminative alternative as well as an overall decrease in latency across phases (see Fig 7). Indeed, these effects were corroborated by the linear mixed effects model which showed a main effect of phase, *F*(1, 9) = 10.21, *p* = .011, and trial type, *F*(1, 9) = 9.44, *p* = .013. Rates of responding to each terminal link stimulus were nearly identical between the two stimuli (see Fig 7) and, although there appeared to be a general decrease in response rates across phases, the main effect of phase, *p* = .104, did not reach statistical significance.

### Discussion

The purpose of Experiment 2 was to assess a second prediction made by the stimulus value hypothesis that when terminal link stimuli are equally predictive pigeons will be relatively indifferent between them. The results of Phases 1 and 2 both showed strong preferences for the Discriminative alternative because its terminal link stimulus had greater predictive value than the Nondiscriminative alternative’s (100% vs. 50% in Phase 1 and 75% in Phase 2). Also consistent with the stimulus value hypothesis was that, when the terminal link stimuli became equally predictive (100% vs. 100%), there was a significant reduced preference for the Discriminative alternative. However, as can be seen in Fig 6, preference for the Discriminative alternative did not decline to indifference (50%) as was found by Smith and Zentall [20]. It is very likely that the training these pigeons had involving a predicted preference for the Discriminative alternative may have maintained that preference in the absence of differential stimulus values. That is, when pigeons are indifferent between alternatives, they may revert to either a spatial bias or, as in the present case, continue to choose a previous bias (the Discriminative alternative). Had these pigeons been trained from the start with equal valued terminal stimuli, the results may have been more similar to previous studies [20, 26].

Similar to Experiment 1, latencies were again shorter for the Discriminative than the Nondiscriminative alternative and were consistent with the choice measure. These results qualitatively support the hypothesis that latencies may reflect decision making processes with this task [7, 23]. When the predictive validity of the Nondiscriminative alternative was increased, one might have predicted that the latencies to that alternative would have shortened (indicative of its increased value) similar to what was seen in Experiment 1, but the latencies did not appear to mirror those trends as predicted by the model. Response rates to the terminal link stimuli again showed no differences, further suggesting they are not useful proxies for measuring preferences in this procedure.

## General Discussion

The purpose of the present experiments was to further test the predictions of the stimulus value hypothesis [3, 8, 20, 21]. This hypothesis suggests that the value of the terminal link conditioned reinforcers will determine the initial link preference relatively independent of their frequency. Thus, when the terminal link stimuli have equal predictive value, the pigeons should be relatively indifferent between them. This prediction was supported in Experiments 1 and 2. In both experiments, the alternative with the terminal link stimulus of greatest predictive value was preferred with decreasing frequencies of the predictive stimulus resulting in little reduction in initial link preference. In Experiment 2, only when the predictive stimuli were of equal value (Phase 3) was there any reduction in preference. Although that preference did not reach indifference and individual differences were present, it is likely that the maintained preference for the Discriminative alternative reflected carry over effects due to the extensive prior training in which the Discriminative alternative was preferred.

The present research also shows that a simplified 2-stimulus design can produce results similar to that of the more complex 3- [17, 20] and 4-stimulus designs [6, 12] and that it is unnecessary to include a stimulus associated with the absence of reinforcement for pigeons. Apparently, including such a stimulus does little to inhibit choice of the suboptimal alternative [14–16]. Thus, this simplified design can be advantageous for testing, what we argue, to be the core mechanism responsible for suboptimal choice: the value of the conditioned reinforcers. The 2-stimulus design may also make it easier to test the stimulus value hypothesis in species that are less visual than the pigeon and have greater difficulty discriminating a larger number of stimuli. Indeed, recent research has used the 4-stimulus design with species such as starlings [7], rats [27], and humans [10] making the present procedure potentially advantageous in this translational process.

This procedure may also serve as a useful animal analog of human gambling because humans predisposed to problem gambling have been hypothesized to seek highly rewarding events [28, 29] and anticipate winning [30] while attending very little to losses. Thus, it is possible that the pigeons’ preference for the predictive stimulus despite losses in reinforcement may be analogous to win seeking by humans when they gamble. Additionally, this procedure (using a 4-stimulus design) has already shown some efficacy in distinguishing humans who gamble from those who do not [10]. Future research should continue to investigate the propensity of reward-predictive cues to potentiate problem gambling behaviors.

### Alternative Theories

Given the recent renewed interest in the suboptimal choice paradigm [7, 8, 16, 20, 24, 27], it is appropriate to consider how competing theories may account for the results of the present experiments. For example, when choice of the Nondiscriminative alternative results in a stimulus associated with reinforcement less than 100% of the time, Vasconcelos, Monteiro, and Kacelnik [7] suggest that the Discriminative alternative will be preferred because the time spent in the presence of a predictive stimulus reduces the time spent in uncertainty. It is worth noting, however, that preference for a Discriminative (suboptimal) alternative does not depend on the terminal link stimulus being perfectly predictive of reinforcement. Indeed, Zentall and Stagner [31] found that, when the predictive validity of the Discriminative alternative’s terminal link stimulus was reduced to 80% predictive of reinforcement, a strong preference remained. Thus, as long as the predictive stimulus following the Discriminative alternative has greater predictive validity than the Nondiscriminative alternative, it should be preferred.

A separate model of suboptimal choice, proposed by Spetch, Belke, Barnet, Dunn, and Pierce [17] and Dunn and Spetch [32], suggests that the Discriminative alternative should be preferred because it signals a reduction in the delay to reinforcement. Conceptually similar to that of Vasconcelos et al. [7], this hypothesis attributes the change from the Discriminative alternative’s initial link to its predictive terminal link as a change from uncertainty to certainty. The associated excitatory strength engendered by this change and the signaled reduction to reinforcement is thus responsible for its preference [24]. Additionally, the preference for the Discriminative alternative should become stronger as the duration of the terminal link increases. This should occur because at shorter terminal link delays primary reinforcement strength would be stronger than the conditioned reinforcement strength whereas at longer delays the immediate conditioned reinforcement strength associated with the appearance of the predictive stimulus should become stronger. This argument has been supported by the effect of inserting a gap between choice of the Discriminative alternative and its predictive stimulus [5, 7]. The effect of the gap markedly decreased its preference, yet a similar effect did not occur when the gap followed the Nondiscriminative alternative that was also followed by a similarly predictive stimulus [5]. The argument is that the gap reduced the delay reduction resulting from choice of the Discriminative alternative but there was no delay reduction following choice of the Nondiscriminative alternative.

This dissociation using the gap procedure led Spetch and colleagues to conclude that it is only the Discriminative alternative’s terminal link stimulus that acts as a conditioned reinforcer. However, such a theory is unable to account for the findings from Smith and Zentall [20] and Stagner, Laude, and Zentall [26] that showed indifference between initial link alternatives when the terminal link stimuli were equally predictive of reinforcement. In both experiments there should have been a preference for the Discriminative alternative but instead there was indifference. The delay reduction interpretation is also challenged by the results of the present experiments because there were no changes in preference for the Discriminative alternative with decreases in the frequency of its predictive stimulus. To be consistent with the theory, this result would require that the excitatory strength attributed to the appearance of the predictive stimulus (which should increase with reduced frequency) to be offset by its reduced primary reinforcement value equally in all three phases of Experiment 1.

Thus, the current and previous research challenges the interpretations of Spetch et al. [17] and Dunn and Spetch [32] with evidence favoring the simpler stimulus value hypothesis. Additionally, although the theory presented by Vasconcelos et al. [7] could be considered in keeping with the results of the present research, it predicts psychological effects beyond those tested here. Indeed, future research may yet show that these competing hypotheses may play a role in suboptimal choice. However, at present we prefer the simpler and more parsimonious stimulus value hypothesis that has, to this point, been effective in predicting suboptimal choice preferences.

### Stimulus Value

We use the term stimulus value to describe these suboptimal choice effects, but this is synonymous and interchangeable with conditioned reinforcement. Mazur [3] originally suggested the strength or value of an alternative is a function of the probability of a delay to the primary reward, shown in Eq 1:

$$V=\sum_{i=1}^{n}P_{i}\left(\frac{1}{1+KD_{i}}\right)$$ (1)

where *V* is the value of the alternative that hyperbolically decreases as the probability, *P*_*i*_, of a delay, *D*_*i*_, occurs between choice and delivery of a reinforcer while *K* is a free parameter that determines the rate of value reduction when a conditioned reinforcer is present [21]. Thus, Mazur suggested in Eq 1 that the value of the terminal link stimulus is a function of summed probabilistically weighted delays of each terminal link.

In the present design, delay to reinforcement was held constant as both alternatives appeared for equal durations (10 s), yet the Discriminative alternative produced a conditioned reinforcer only when food was scheduled to be delivered; this makes the probability of delay always 10 s. Using Eq 1 and setting *K* to 1, this approximates the value for the predictive stimulus at 0.091 for that trial (for Experiment 1). Alternatively, the Nondiscriminative alternative always produced a conditioned reinforcer that was only sometimes followed by food. As an example, when reinforcement occurred on the first choice, the value of the nonpredictive stimulus would also be 0.091; however, because the probability of reinforcement was 50% reinforcement might not have occurred until a second or third choice (25% and 12.5% likelihood, respectively), and, according to Mazur’s theory, the 20- and 30-s delays would devalue the nonpredictive stimulus to 0.012 and 0.0095, respectively, for that trial. Summing together these three hypothetical trials, it can be seen that the predictive stimulus holds a greater value (0.273) than the nonpredictive stimulus (0.113). Eq 1 therefore predicts an ordinal preference for the Discriminative alternative until the final phase of Experiment 2, similar to the results of the present experiments.

Alternatively, Grace’s contextual choice model (CCM [33]), a temporally constrained derivation of the concatenated matching law for concurrent chain choice [34–36], also suggests that initial link responding is a function of relative conditioned reinforcement rate, yet it allows for a dissociation between primary and conditioned reinforcement not explicitly recognized in Eq 1. However, in the present design, as the time spent in the initial links was the same between alternatives, the scheduled temporal context expressed in CCM is constant between alternatives, simplifying the CCM to Eq 2:

$$\frac{B_{1}}{B_{1}+B_{2}}=\frac{100}{1+{\left(\frac{R_{1}}{R_{2}}\right)}^{s_{r}}{\left(\frac{CR_{1}}{CR_{2}}\right)}^{s_{cr}}}$$ (2)

where the relative rate of behavior on one alternative (i.e., preference for the Discriminative alternative), *B*_*1*_, is proportional to the multiplicative combination of both its scheduled rate of primary reinforcement, *R*_*1*_, and conditioned reinforcement (terminal link value), *CR*_*1*_, relative to the alternative (i.e., the Nondiscriminative alternative), *R*_*2*_ and *CR*_*2*_. Each of these relative ratios is then raised to a parameter value indicating either the relative primary, *s*_*r*_, or conditioned, *s*_*cr*_, reinforcement sensitivity. Eq 2 therefore predicts that initial link preference is a function of an individual’s sensitivity to relative primary (*s*_*r*_) and conditioned (*s*_*cr*_) reinforcement, with greater sensitivity values indicating greater influence over behavior either towards preferring (if positive) or not preferring (if negative) the Discriminative alternative.

**Fig 8:**
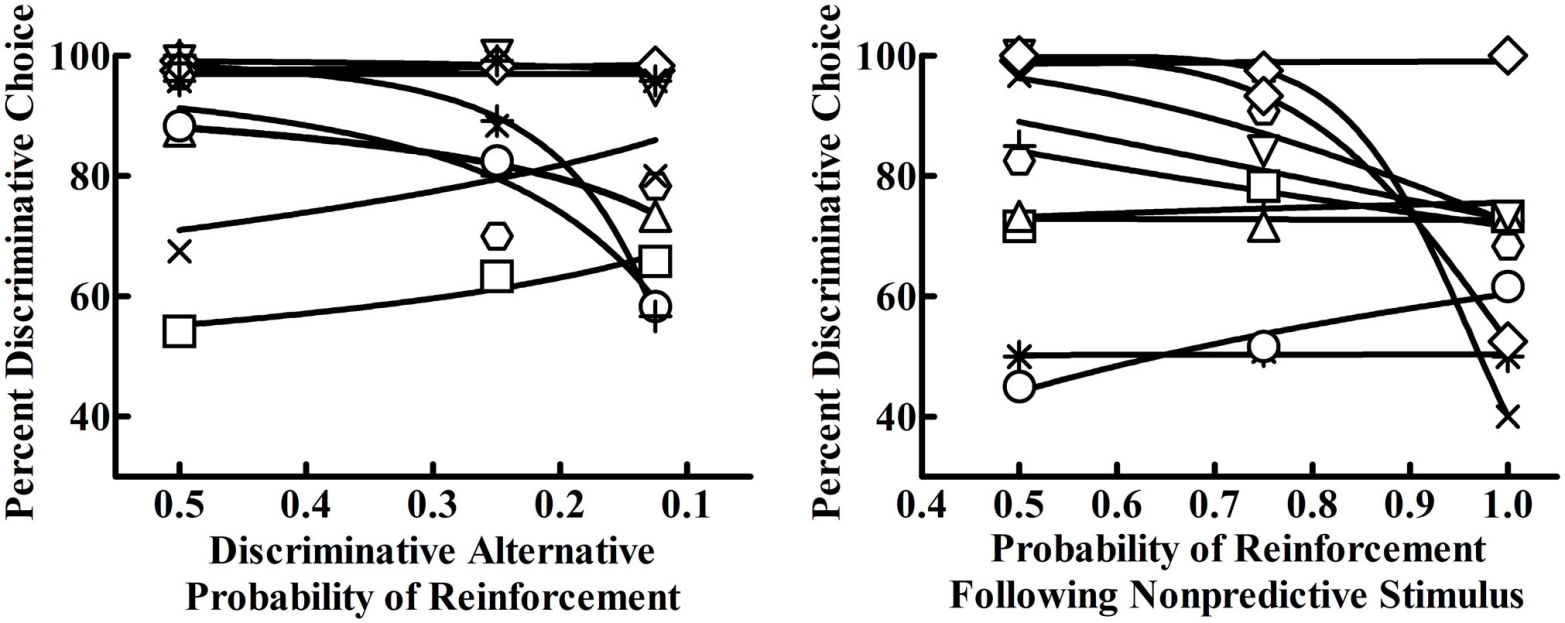
Individual fits of Eqs 3 and 4 on the average percent Discriminatve alternative choice as a function of Phases 1–3 in Experiment 1 (left) and 2 (right).

Eq 2 generally fit well in both experiments (see Fig 8). While individual differences were apparent, sensitivity to primary reinforcement was relatively low in Experiment 1 (*s*_*r*_ = 0.55, *SEM* = 0.43) and Experiment 2 (*s*_*r*_ = 2.73, *SEM* = 1.26), while sensitivity to conditioned reinforcement was higher (*s*_*cr*_ = 3.74, *SEM* = 0.60; *s*_*cr*_ = 4.14; *SEM* = 1.03). Thus, Eq 2 suggests that, while there was some sensitivity to changes in relative primary reinforcement, sensitivity to conditioned reinforcement mostly determined initial link preference in both experiments.

One limitation to the above approach in accounting for the data in Experiment 2 is the confounding of primary and conditioned reinforcement, as both the nonpredictive stimulus’ predictive validity for food and primary reinforcement were increased simultaneously. To better dissociate primary from conditioned reinforcement mechanisms, future research should seek to manipulate primary and conditioned reinforcement independently, as was done in Experiment 1. For example, another way to conduct Experiment 2 may have been to equate conditioned reinforcement value (while isolating primary reinforcement effects) and manipulate the Nondiscriminative alternative’s nonpredictive stimulus through its predictive validity and frequency of appearance. To keep primary reinforcement at approximately 50%, the nonpredictive stimulus in Phase 1 of Experiment 2 could have been increased to 75% predictive of food but only appear 67% of the time and then to 100% predictive of food but appearing only 50% of the time. This manipulation would better isolate the two terms in Eq 2 and possibly provide better fits that attribute greater sensitivity to the conditioned reinforcement values.

Reinforcement is multidimensional in nature, meaning that many factors can alter conditioned reinforcers and by extension can affect suboptimal choice. For example, Mazur [3] has shown that differential delays to reinforcement can influence initial link choice. Additionally, differential magnitude of reinforcement may systematically alter the conditioned reinforcing value of the terminal link stimuli. For example, each alternative may be followed by a conditioned reinforcer that predicts food 100% of the time, but one alternative may deliver 10 pellets of food while the other delivers 3 [2, 37], making the relative predictability of the terminal link stimuli (*CR*_*1*_ and *CR*_*2*_) the same, but large differences in primary reinforcement magnitude that can also generate a suboptimal choice effect. While the present experiment only assessed primary and conditioned reinforcement (the conditioned reinforcer’s predictive validity for food), future research may incorporate additional dimensions of reinforcement (such as delay or magnitude). For each of these different reinforcer dimensions, Eq 2 can be modified [33] to incorporate those dimensions being manipulated in Eq 3:

$$\frac{B_{1}}{B_{1}+B_{2}}=\frac{100}{1+{\left(\frac{R_{1}}{R_{2}}\right)}^{s_{r}}{\left[{\left(\frac{X_{1}}{X_{2}}\right)}^{s_{x}}{\left(\frac{CR_{1}}{CR_{2}}\right)}^{s_{cr}}\right]}^{{\left(\;\frac{T_{t}}{T_{i}}\right)}^{k}}}$$ (3)

where the sensitivities to a particular reinforcer dimension (*X*_*1*_ and *X*_*2*_), like magnitude or delay to reinforcement, are raised to a sensitivity parameter (*s*_*x*_).

Eq 3 is thus similar to Eq 2 in that initial link preferences are a multiplicative function of primary and conditioned reinforcement; however, there are two important differences. First, dimensions of reinforcement such as delay and magnitude (expressed as variable *X*) are considered part of terminal link value (that is, they affect conditioned rather than primary reinforcement). Second, the combined terms for terminal link values are further multiplied by the ratio of terminal link durations, *T*_*t*_, to initial link durations, *T*_*i*_, with *k* as a scaling factor. Eq 3 therefore makes the novel prediction that the suboptimal preference shown in the present experiments may have been due in part to the increased time in the terminal links relative to the initial links. Although delays were not manipulated here, a testable prediction to reduce suboptimal preferences would be to increase the time spent in the initial links relative to the terminal links; that is, according to Eq 3, increasing the initial link duration, relative to the terminal link duration, should reduce the sensitivity to conditioned reinforcement. Such a prediction has been assessed previously [32], but its interpretation is complicated due to the use of a spatial discrimination as previously discussed in the introduction. Each of the above models (or their combination [38]) may have utility in describing suboptimal choice, but there is insufficient data at present to conduct clear tests between them. Future research should assess multiple conditions, independently manipulating a single dimension, in order to better identify the mechanisms responsible for these suboptimal preferences.

## Conclusion

The present research sought to test the predictions of the stimulus value hypothesis. In line with these predictions, the present research found that the terminal link stimulus with the greatest predictive validity for reinforcement predicted initial link preference. This preference remained despite substantial reductions in the frequency of the predictive stimulus and preference declined primarily when predictive stimuli of equal value followed both choice alternatives. The present suboptimal choice procedure may thus serve as a simplified version that can readily be used to study suboptimal choice in other species. Several theories and models attempting to account for these data were also offered and discussed, yet the currently available evidence seems best predicted by the simpler stimulus value hypothesis which may have implications for human gambling behaviors.

## Supporting Information

S1 Dataset Experimental Data. This file contains all data used in the statistical and mathematical models of the present experiments.
(XLSX)
